# The deficiency of adenosine deaminase type 2-results of therapeutic intervention

**DOI:** 10.1186/1546-0096-13-S1-O40

**Published:** 2015-09-28

**Authors:** A Ombrello, D Stone, P Hoffmann, A Jones, B Barham, K Barron, W Flegel, S Sheldon, Q Zhou, M Hershfield, I Aksentijevich, P Kumar, D Kastner

**Affiliations:** 1 NIH, NHGRI, Bethesda, USA; 2 NIH, NIAID, Bethesda, USA; 3 NIH, Department of Transfusion Medicine, Bethesda, USA; 4 Duke, Department of Biochemistry, Durham, USA; 5 NIH, Pharmacy, Bethesda, USA

## Introduction

The deficiency of adenosine deaminase type 2 (DADA2) is a recessively inherited condition caused by mutations in *CECR1*. Patients present with recurrent fevers and evidence of vasculitis/vasculopathy, including livedo racemosa, lacunar strokes, polyarteritis nodosa, endothelialization of the hepatic sinusoids with portal hypertension, and active colitis. There is no recombinant form of ADA2 and thus we attempted a) exogenous replacement of ADA2 via fresh frozen plasma (FFP) and b) suppression of the inflammatory response using anti-tumor necrosis factor (anti-TNF) therapy. This abstract documents 22 months of clinical treatment in the NIH DADA2 cohort.

## Objectives

a) To determine if FFP infusion is safe and if it would result in sustainable increased ADA2 levels; b) assess clinical and laboratory response to anti-TNF agents.

## Patients and methods

***FFP safety and pharmacokinetics:*** To assess the safety of FFP infusion, 3 DADA2 patients were admitted to the NIH Clinical Center for 5 consecutive days of FFP infusions. Serial ADA2 levels were drawn and FFP volumes were increased, barring adverse effects, each day. Subsequently, the patients were re-admitted and administered a single 100 mL FFP dose. Blood samples were drawn for ADA2 analysis.

***Anti-TNF therapy:*** After comprehensive evaluation, patients were started on anti-TNF therapy. Patients underwent follow-up evaluation after approximately one year on treatment. Follow-up procedures were completed as needed.

## Results

***Pharmacokinetic results:*** Utilizing the observed increase in ADA2 levels in patients from the pre- and post-infusion samples over the 5 days of FFP infusions, a volume of distribution for ADA2 of approximately 1100 ml (range: 605-2207 ml) was calculated. When serial ADA2 sampling was conducted in the subsequent admission, the median terminal half-life was approximately 6.4 hours (range: 4.95-8.95 hours).

***Anti-TNF results:*** 12 patients were treated with anti-TNF agents. The median time on therapy was 10 months (IQR: 8.5-19). There were no significant new disease complications (strokes, GI ischemia, worsening portal hypertension). Inflammatory markers stabilized in all cases and anemia improved. One patient with documented portal hypertension and esophageal varices had variceal resolution after 12 months of therapy. The cutaneous PAN lesions improved but there was persistent livedo racemosa.

## Conclusion

Although the administration of FFP caused a transient increase in serum ADA2 levels, the short half-life would necessitate that large volume infusions (>200-300 ml) be given at least daily, rendering this treatment unfeasible. There has been dramatic clinical and laboratory improvement on anti-TNF agents.

